# High Aspect Ratio Silver Nanogrids by Bottom-Up Electrochemical Growth as Transparent Electrode

**DOI:** 10.1021/acsaom.4c00037

**Published:** 2024-03-13

**Authors:** Yorick Bleiji, Andrea Cordaro, Stefan W. Tabernig, Esther Alarcón-Lladó

**Affiliations:** Center for Nanophotonics, AMOLF, Science Park 104, 1098 XG Amsterdam, The Netherlands

**Keywords:** electrodeposition, silver, nanowire grid, transparent electrode, high aspect ratio

## Abstract

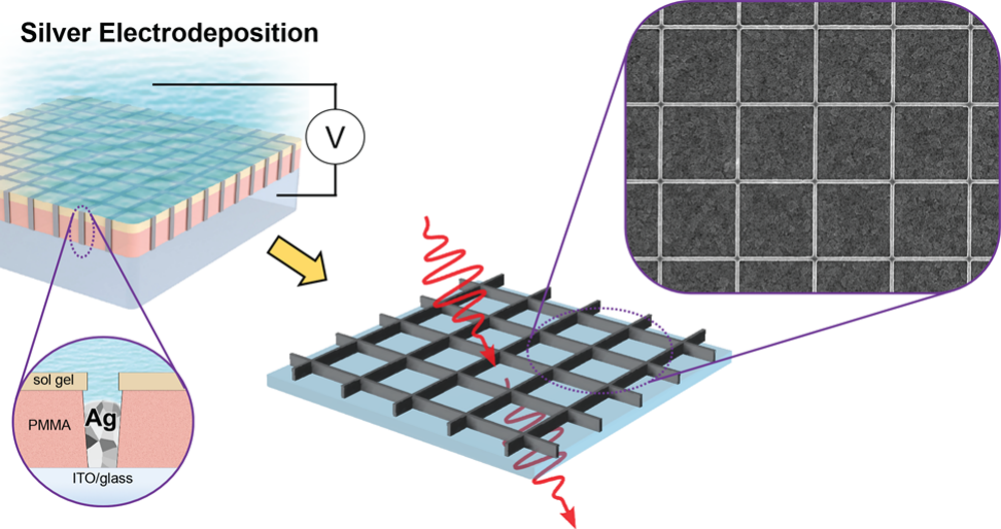

A scalable selective-area electrochemical method is reported for the fabrication of interconnected metal nanostructures. In this work, the fabrication of silver nanowire grids for the application of transparent electrodes is explored. The presented method is based on a through-the-mask electrodeposition method, where the mask is made by using substrate conformal imprint lithography. We find that the nucleation density of the silver nanoparticles is the key parameter for successful homogeneous void-free filling of the template. We independently controlled the density of the silver nuclei and their growth by using a double potential pulse. The silver nanowire grids show high transmission (95.9%) and low sheet resistance (as low as 3.7 Ω/sq), resulting in a superior figure of merit (FoM). Due to the bottom-up nature of this technique, arbitrarily high aspect ratio nanowires can be achieved, therefore decreasing the sheet resistance without affecting transmittance and carrier collection. The presented method can be generalized to the large-area nanofabrication of any well-defined nanostructure design of any metal transparent electrode for multiple applications.

## 1 Introduction

Transparent electrodes (TEs) are critical components in numerous optoelectronic devices, such as displays,^1^ smart windows,^2^ touchscreens,^3^ (organic) light-emitting diodes (LEDs),^4^ and solar cells.^5−9^ As technology advances, high-quality TEs are nowadays required to reduce the power consumption in efficient optoelectronic devices. In solar cells, high-quality TEs are essential to minimize power losses, particularly in cells with the top layer having a short carrier diffusion length, such as amorphous silicon, silicon heterojunction (SHJ), perovskite, copper indium gallium diselenide (CIGS), or organic cells.^5,9−11^ In most commercial applications, metal oxides, indium tin oxide (ITO) in particular, have been most widely used due to their high transmittance, good conductivity, and complementary metal–oxide–semiconductor (CMOS)-compatible fabrication.^7,9^ However, indium is a rare element that should be replaced,^12^ and after decades of optimization, ITO has reached its fundamental limits for transparency and sheet resistance, where the two are linked by the ITO thickness.^13,14^

In recent years, metal nanowire (NW) networks have received strong attention due to their excellent conductivity and mechanical flexibility, which broadens the range of TE applications to flexible optoelectronics.^15^

A wide variety of NW network geometries have been proposed in the context of solar cell applications, which can be designed to increase device performance by making use of nanophotonic effects such as (plasmonic) light trapping^5,16,17^ or spectral splitting for tandem devices.^18,19^ Metal NW networks can be fabricated using solution-based processes that enable high-throughput large-scale manufacturing.^20^ Random networks have been demonstrated by colloidal synthesis of NWs followed by drop casting.^21−24^ As-deposited networks often result in poor electrical uniformity throughout the electrode, which is improved by subsequent thermal annealing.^21−24^ Similar to ITO, colloidal-based NW networks suffer from a trade-off between transparency and resistance. Increasing the NW radius or density is used to reduce sheet resistance but reduces transparency at the same time.

On the other hand, increasing the aspect ratio of the NW cross section offers the possibility to break this transparency–resistance trade-off. A reliable approach to fabricating arbitrary NW cross sections is the use of lithography. Initial work demonstrated the high potential of periodic Ag NW networks by using e-beam evaporation and lift-off. While post-annealing is not necessary for a low junction resistance, this method still suffers from complex and expensive fabrication and is limited to small NW heights (i.e., low aspect ratio of the NW cross section) to avoid lift-off issues.^5,14,25,26^

On the contrary, template-assisted electrodeposition combines solution-based processing with well-defined bottom-up patterning without the need for lift-off. In template-assisted electrodeposition, tailor-made trenches in an insulating mask are conformally filled from the bottom up. Metal microwire grids have been demonstrated by this method, where the original template mask can even be reused multiple times by carefully peeling off the metal grid.^27,28^ However, as the trenches are scaled down to the nanoscale, achieving uniform, void-free filling over a large area without a seed layer remains a challenge.^29^

In this work, we demonstrate the fabrication of highly performing TEs by combining substrate conformal imprint lithography (SCIL) with electrodeposition as a scalable and sustainable method for the large-scale fabrication of sub-100 nm metal nanostructures. It has been shown that SCIL can be used for imprinting both rigid and flexible substrates up to an area of a 200 mm wafer with sub-10 nm resolution.^30^ First, SCIL is used to make an insulating template consisting of a grid of deep nanotrenches (80 nm in width and up to 300 nm in depth). Subsequently, these nanotrenches are selectively filled with silver by electrodeposition. Using this method, we obtain NW grids with tailored aspect ratio wires (a height/width ratio of up to ∼3.5). We show that the high aspect ratio Ag NW grids have a superior figure of merit (FoM), where we improve the sheet resistance without affecting transmittance or carrier collection. This template-assisted electrodeposition method is, therefore, a more sustainable method, enabling wafer-scale manufacturing of highly performing TEs.

## 2 Experimental Section

### 2.1 Preparation of the Mask

Substrate conformal imprinting (SCIL) was used for the fabrication of the mask. ITO substrates (KinTec, 10 and 100 Ω/sq, 25 × 25 mm^2^) were cleaned by brushing them with soap and sonicated for 10 min in ultrapure water, 10 min in acetone, and 5 min in isopropanol. The ITO substrates were cleaned for 1 min by using an oxygen plasma (Oxford Plasmalab 80+, 50 W, 5 mTorr) to activate the surface. The poly(methyl methacrylate) (PMMA) (MW = 950 A8 1:1 anisole, Kayaku Advanced Materials, Inc.) spacer was spin-coated onto the ITO substrate at 2000 rpm for 45 s and baked at 150 °C for 2 min. The surface of PMMA was activated using a 30 s oxygen plasma etch (Oxford Plasmalab 80+, 50 W, 5 mTorr). The sol–gel (T1100, SCIL Nanoimprint Solutions) was spin-coated on the PMMA layer at 2000 rpm for 10 s. The polydimethylsiloxane (PDMS) stamp was pushed into the sol–gel layer and removed after 6 min of curing at room temperature. The residual layer of the sol–gel at the bottom of the imprint was removed using a reactive ion etch using HF_3_/Ar (Oxford Plasmalab 80+, 67 W, 15 mTorr) for 2 min and 30 s, and the PMMA was etched using O_2_ plasma (Oxford Plasmalab 80+, 200 W, 5 mTorr) for 228 s. The final depths of the trenches were between 300 and 350 nm.

### 2.2 Electrochemical Superfilling of the Trenches with Silver

A custom-built PEEK cell of 24 mL volume was used, using a standard three-electrode configuration (see Figure S1 in the Supporting Information). A Pt disc (exposed area of 3.08 cm^2^) was used as the counter electrode, and a Ag/AgCl electrode (leakless miniature ET072, EDAQ) was used as the reference electrode. Before the start of each experiment, the miniature reference electrode was calibrated against a saturated Ag/AgCl reference electrode (XR300, Hach). All experiments were performed using an SP-300 BioLogic potentiostat.

A commercial silver plating solution (Clean Earth Solutions, 45.220 g) was used for the electrochemical superfilling of the nanotrenches. The double pulse method was used to control the nucleation density and growth rate of the wires independently. A nucleation pulse of *E*_n_ = −0.96 V vs Ag/AgCl was applied for *t*_n_ = 750 ms, followed by a growth pulse of *E*_g_ = −0.06 V vs Ag/AgCl with varying growth time *t*_g_ from 14 to 550 s.

### 2.3 Sheet Resistance Measurements

The sheet resistance *R*_sh_ of the samples was measured directly before and directly after the electrochemical deposition of silver using the van der Pauw method. Four gold pins located at the bottom of the electrochemical cell were used to perform a four-point probe resistance measurement using an SP-300 BioLogic potentiostat. A cloverleaf type of configuration was used, where four scratches were made on the ITO substrate to make sure that the current ran through the center region of the sample. For a more detailed description and calculation of the sheet resistance, see section S2 of the Supporting Information.

### 2.4 Mask Removal

The PMMA/sol–gel mask was removed by submerging the samples (vertically oriented) in 40 °C acetone for >15 min while stirring the solution with a magnetic stir bar.

### 2.5 Transmission Measurements

The transmission spectra of the samples were obtained by using a PerkinElmer UV/vis/NIR Lambda 750 integrating sphere. The transmission spectra of the bare Ag NW grids were obtained by dividing the Ag NW + ITO spectra by the ITO reference spectra. The average transmission was obtained by taking a weighted average using the AM1.5 spectrum. For more details, see section S3 of the Supporting Information.

### 2.6 Morphological Characterization

Morphological and structural characterization of the Ag NW grids was performed using an FEI Verios 460 scanning electron microscope (SEM) that operated at 5 kV and 100 pA, using a working distance of 4 mm. An edge detection Python algorithm was used to extract the width from the SEM images. First, a threshold was used to convert the SEM images to binary images. Next, the width in pixels of the nanowires was extracted line by line for both the vertical and horizontal directions and transformed into the width in nanometers by using the pixel size. The average width was obtained by extracting the expected value from a Gaussian fit to the combined width distributions of the horizontal and vertical nanowires. The error in the width was taken to be the standard deviation of the Gaussian fit. Topographical maps were obtained with atomic force microscopy (AFM) using a Bruker Dimension Icon instrument and a ScanAsyst-Air probe (Bruker, nominal tip radius of 2 nm). The height of the wires was extracted by fitting a Gaussian distribution to the masked area corresponding to the wires in the AFM images.

### 2.7 X-ray Diffraction

X-ray diffraction (XRD) was performed on Ag NW grids on thin ITO substrates (100 Ω/sq) by using a Bruker D2 Phase diffractometer. The Cu K_α_ irradiation was operated at 30 kV and 10 mA. The substrates were scanned between 2θ = 36° and 40° with a 0.008° increment and a dwell time of 0.1 s. More than 40 scans were obtained to increase the signal-to-noise ratio. The XRD scans were corrected by subtracting the background and removing the K_α2_ peak.

## 3 Results and Discussion

**Figure 1:**
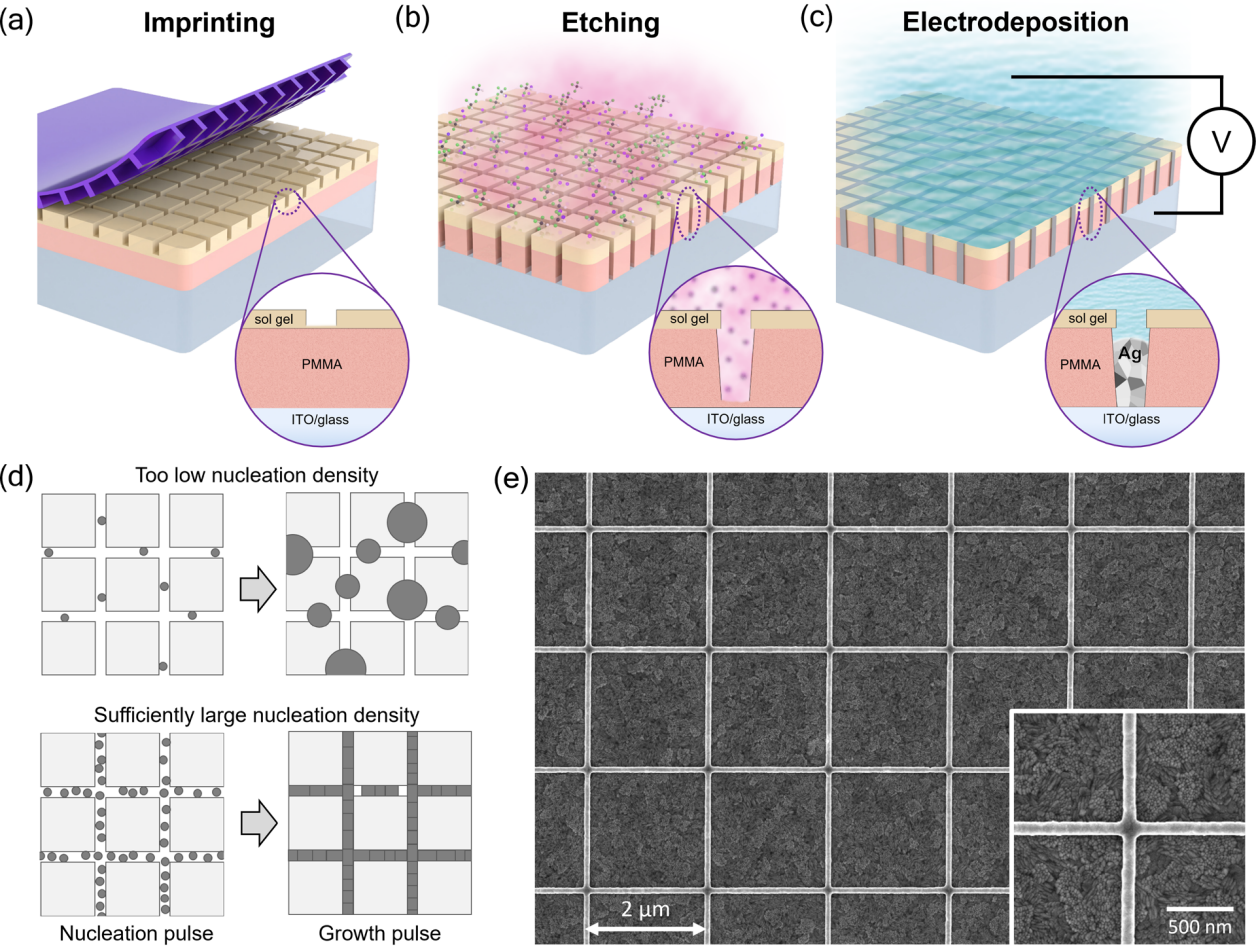
(a) Schematic representation of the SCIL imprinting procedure. (b) Schematic representation of the RIE of the mask to etch the residual layer of the sol–gel and transfer the pattern to the PMMA layer. (c) Schematic representation of the electrochemical filling of the trenches. (d) Schematic representation of the influence of the nucleation density. The top and bottom rows show the effect of a too low and a sufficiently large nucleation density, respectively. (e) SEM image of a typical template-assisted electrodeposited Ag NW grid having a pitch of 2 μm. The inset shows the crossing of two NWs in more detail.

The fabrication of the high aspect ratio Ag NW grids is schematically shown in Figure 1a–c. First, a PMMA/sol–gel mask on an ITO substrate was made using substrate conformal imprint lithography (SCIL). Note that ITO was used here as a proof of concept, but any other conductive substrate can be used, including doped silicon (see section S4 of the Supporting Information). Alternatively, subsequent peel-off and transfer of the nanowires to another substrate may be considered.^27,31^ To pattern the mask, the sol–gel layer is imprinted with a PDMS stamp with the desired grid geometry and is then transferred to PMMA by using a reactive ion etch (RIE) until the substrate is exposed. The PMMA/sol–gel stack determines the final depth of the trench, which in this work is typically around 300–350 nm (aspect ratio of ∼4). Here, we used two different stamps with square grids of 80 nm-wide nanowires and a pitch *L* of either 2 or 4 μm. Both stamps have an imprint area of 2.0 × 2.0 cm^2^.

After imprinting, the nanosized trenches are filled with silver using electrochemical deposition using a commercial Ag plating solution (see the Experimental Section). The growth area is restricted to an area of 0.95 cm^2^ by the O-ring used in the electrochemical cell.

To achieve high-quality, homogeneous, and void-free NW grids, we use the double potential pulse technique to independently control the nucleation density and the grain growth rate.^32^ More details on the nucleation mechanism of silver on ITO substrates can be found in our previous work.^33^ For a complete filling, the nucleation density must be sufficiently large (>2 × 10^9^ cm^–2^) such that the coalescence thickness is smaller than the depth of the trench, as schematically represented in Figure 1d. On nonwetting substrates, a high nucleation density is achieved by applying a high overpotential (*E*_n_ = −0.96 V) over a short period of time. Once the grids are filled with small Ag nuclei, a small overpotential (*E*_g_ = −0.06 V) is applied to slowly and uniformly grow the nuclei until they coalesce into a grid. The height of the grid is then determined by the duration of the growth pulse.

An SEM image of a typical electrochemically grown Ag NW grid is shown in Figure 1e. The grid is highly uniform over large areas (see section S5 of the Supporting Information). The inset of Figure 1e shows that the edges of the individual wires are straight and well-defined, suggesting a highly conformal filling (for more evidence of conformal filling, see section S6 of the Supporting Information). We note that in some cases, small voids can be found at the bottom of the grid owing to the limited density of Ag nuclei. More interestingly, from the close-up SEM image, one can also see that the intersections of the individual wires consist of a continuous deposit. This suggests that no additional resistance is expected at the junctions.

**Figure 2:**
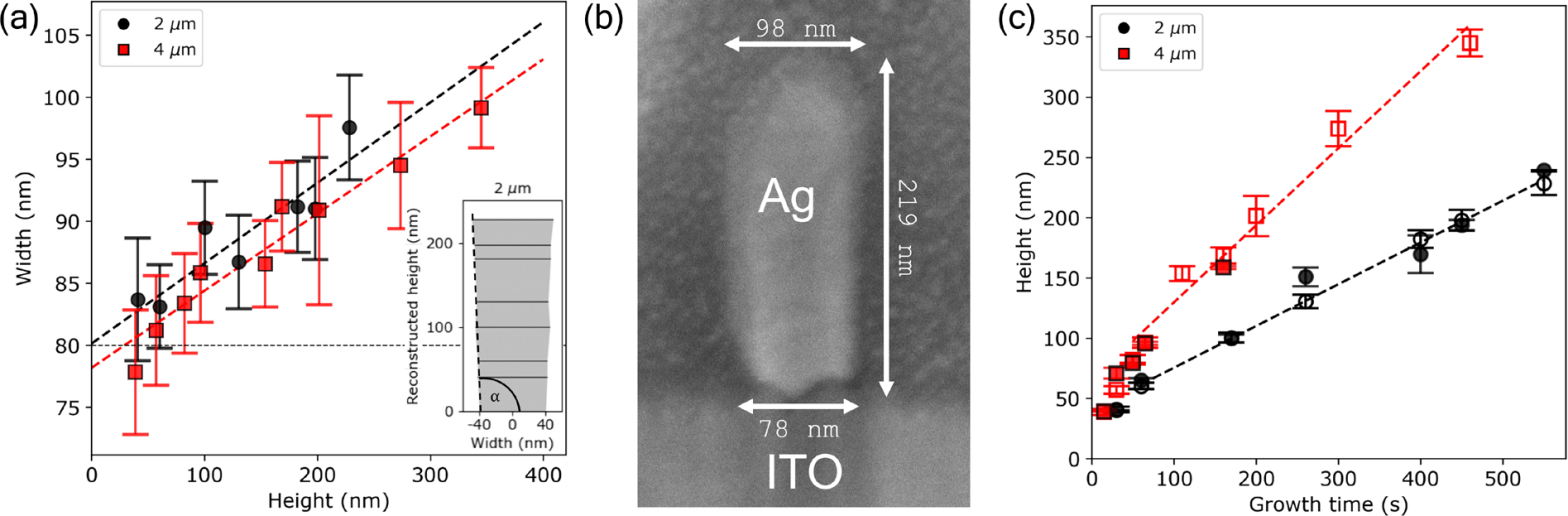
(a) Width of the wires as seen from the top (SEM) vs the grid height obtained from the transferred charge for a pitch of 2 (black circles) and 4 μm (red squares). The inset shows the reconstructed height profile for the 2 μm pitch, including the definition of the inner angle α. The error bars correspond to the standard deviation of the Gaussian fit to the width distribution obtained from the SEM images. (b) Cross-sectional SEM image of a Ag NW grid having a pitch of 2 μm, a height of 219 nm, a top width of 98 nm, and a base width of 78 nm. The inner angle α between the ITO substrate and the Ag NW is 92.6°. (c) Height of the Ag NW grids vs the growth time for a pitch of 2 (black circles) and 4 μm (red squares). The height obtained from AFM and the transferred charge are represented by closed and open markers, respectively. The error on the height obtained from the transferred charge method is propagated from the error on the width, and the error on the height obtained from AFM is the standard deviation of the Gaussian fit to the height distribution.

The cross-sectional SEM image of a NW is shown in Figure 2b. Due to the nonperfect anisotropic plasma etch of the PMMA layer, the trench has a slight trapezoidal profile (i.e., narrow at the bottom, wide at the top, and a characteristic inner angle of α > 90°) that is followed by the Ag filling. Thus, the width extracted from the SEM images by an edge detection algorithm always results in a value that is slightly larger than the nominal width from the stamp (80 nm), and this value increases as the grid grows taller.

The SEM width as a function of height is shown in Figure 2a. We find a clear linear increase in width as a function of height, consistent with the trapezoidal trench profile. The reconstructed Ag NW grid height profile from the width-vs-height measurements is shown in the inset of Figure 2a. The inner angle α of the reconstructed height profile is calculated to be 93.8 ± 1.4° and 93.7 ± 0.8° for the 2 and 4 μm samples, respectively, which is highly consistent with that extracted from the cross-sectional SEM image (α_SEM_ = 92.6°). Extrapolating the width to zero height, we find that the base width of the trapezoid is 80 ± 3 and 78 ± 3 nm for the 2 and 4 μm samples, respectively, which agrees well with the nominal width of the stamp of 80 nm.

To assess the growth rate and the effect of the grid geometry, we plot the grid height as a function of growth time, as shown in Figure 2c. The height of the electrochemically deposited Ag NW grids was determined using two methods. The first method calculates the height based on the total charge transferred during the electrochemical deposition. Here, we assume a Faradaic efficiency of 100% for the Ag deposition and homogeneous growth across all the electrochemical active areas; thus, it serves as an upper limit for the height (for more details, see section S7 of the Supporting Information). The second method is using atomic force microscopy (AFM), for which the PMMA/sol–gel mask was removed. Mask removal is unfortunately not easy for tall NWs (>200 nm). For samples where lift-off was unsuccessful, the height is only estimated from the integrated electrical charge during electrochemical deposition.

Figure 2c shows that both the height from AFM and that from the transferred charge agree well with each other (validating the near 100% Faradaic efficiency and homogeneous deposition) and scale linearly with growth time. At the early stages of growth (growth time < 50 s), the height increases rapidly with time, indicative of the still 3D diffusion-limited growth of individual nuclei. Under this condition, homogeneous growth does not hold, and there might be a higher discrepancy between the height values obtained from the two methods.

Once coalescence is reached (growth time > 50 s), the growth rate (given by the height vs time slope) stabilizes to a constant value, which is highly dependent on the geometry of the grid. Note that under the same growth conditions, the growth rate for the 4 μm pitch samples is almost twice as large compared to that of the 2 μm samples (0.64 and 0.35 nm/s, respectively). The increased growth rate in the sparser grid is explained by the reduced ion competition between neighboring grid lines. In either case, we can tune the aspect ratio of the tailored NW grid from ∼0.5 up to ∼3.5, within a maximum growth duration of 500 s. Knowing the trench geometry, the highly reproducible and homogeneous growth enables in situ monitoring of the grid height by using the total transferred charge.

Now, we focus on the functional performance of the NW grids as TEs. First, we investigated the electronic characteristics. We focus on the resistivity ρ, as it is important not only for the electronic performance of the electrodeposited grids but also for assessing material quality. We obtain the resistivity from the sheet resistance of the NW grid *R*_sh_^Ag^ by using ρ = *R*_sh_^Ag^ × *h*_eff_, where *h*_eff_ = *hw*/*L* is the effective thickness of an equivalent Ag film. Here, *h* is the height of the NW obtained from the charge measurement, *w* is the average width of the NW, and *L* is the pitch of the grid. *R*_sh_^Ag^ is, in turn, obtained by measuring the sheet resistance of the ITO substrate and ITO + Ag NW grids using the van der Pauw configuration just before and after the electrochemical filling of the trenches, respectively (see the Experimental Section for more details). The two contributions (ITO and Ag grid) are decoupled by assuming two parallel resistors. For more information on the measurement and sheet resistance calculation, see section S2 of the Supporting Information.

**Figure 3:**
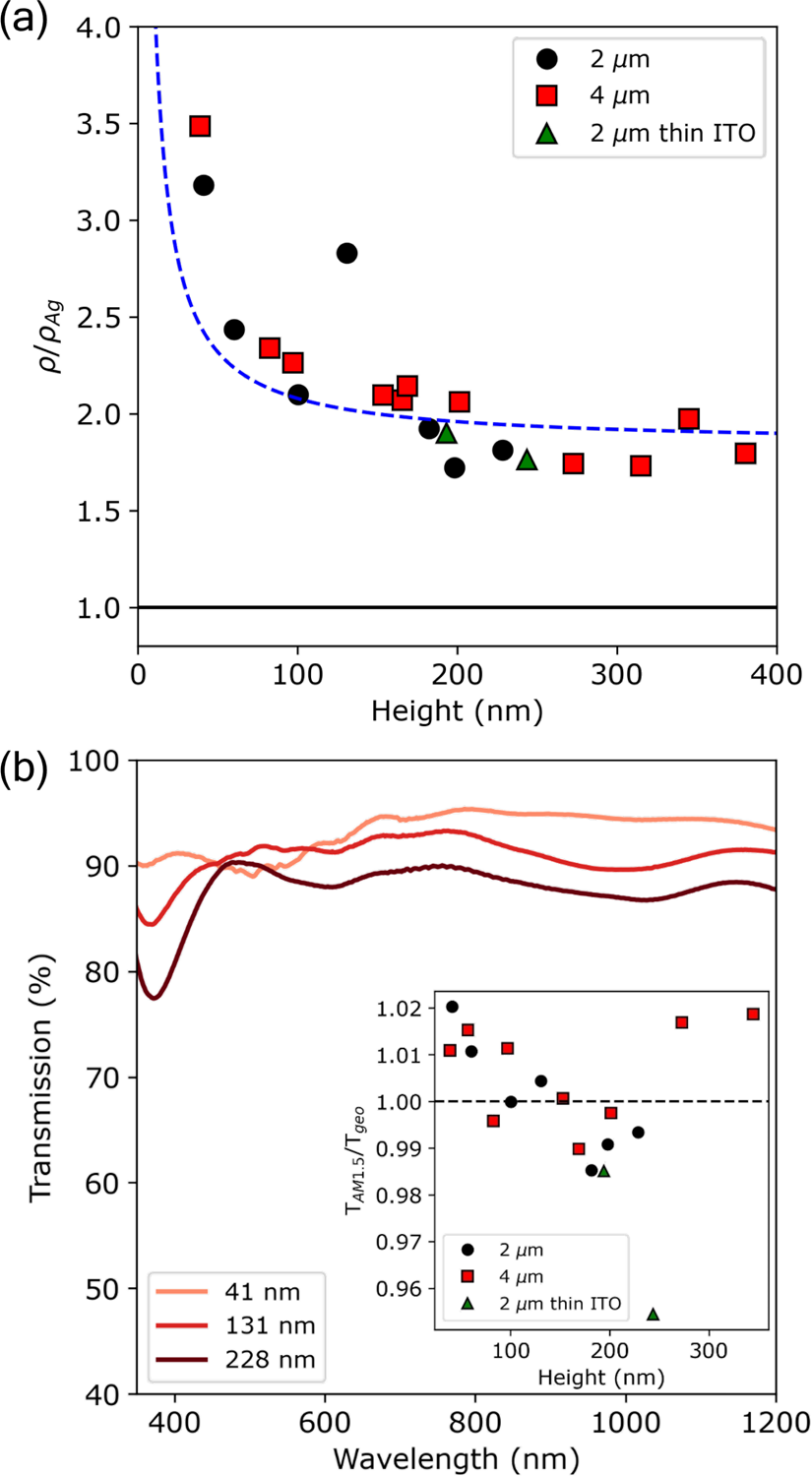
(a) Normalized resistivity vs the height of the Ag NW grids as obtained from the transferred charge for a pitch of 2 (black circles: thick ITO, green triangles: thin ITO) and 4 μm (red squares). The dashed blue line represents the best fit to the electron scattering model using the parameters λ = 58 nm, *w* = 94 nm, R = 0.20, *p* = 0, and *d* = 32 nm. (b) Normalized transmission spectra for a pitch of 2 μm for three different heights (41, 131, and 228 nm). The inset shows the ratio of the AM1.5G weighted transmittance spectra *T*_AM1.5G_ by its geometrical shading *T*_geo_.

Figure 3a shows the normalized resistivity as a function of the Ag NW grid height. We find that the resistivity of all samples follows the same monotonic increase with decreasing height, irrespective of pitch, which is consistent with the fact that electrons in smaller NWs will suffer from increased surface scattering. As a check, we consider the model for the resistivity that includes both inelastic scattering at the surface and scattering at grain boundaries, described by the models of Fuchs and Sondheimer^34,35^ and Mayadas and Shatzkes,^36^ respectively. The combined model results into^37,38^
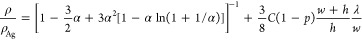
1where ρ_Ag_ is the bulk resistivity of silver (1.59 μΩ cm),  is a scattering factor, *d* is the average grain diameter, λ is the electron mean free path (52–58 nm for Ag),^20,39,40^*R* is the electron reflectivity coefficient (e.g., the fraction of electrons that are scattered by grain boundaries between 0 and 1), and *C* is a geometrical constant, which is 1.2 for nanowires with a rectangular cross section.^38,41^ The parameter *p* is the fraction of electrons that are specularly scattered on the NW surface.

To determine the average grain diameter *d*, we use the position and full width at half-maximum (fwhm) of the Ag(111) XRD peak in combination with the Scherrer equation (see section S8 of the Supporting Information).^20,42,43^ Due to the overlap of the Ag(111) peak with that of ITO, we have grown additional samples on thinner ITO substrates (*R*_sh_ = 100 Ω/sq). The Ag NW grids grown on thin ITO substrates are represented by green triangles in Figure 3a and show comparable resistivity values for the given grid height.

From the XRD analysis, we obtain an average grain diameter *d* of 32 ± 2 nm, which is much smaller than expected from the nucleation density (i.e., the average distance between nuclei is ∼130 nm for a nucleation density of ∼7 × 10^9^ cm^–2^). This result suggests that renucleation takes place during growth, resulting in a smaller average grain diameter. Kung et al. found a similar average grain diameter using the same electrolyte for the electrodeposition of Ag NWs.^42^

Using the average grain diameter from XRD and the average NW width, eq 1 is fitted to the resistivity data in Figure 3a (represented by the blue dashed line), with fit parameters being *R* = 0.20 and *p* = 0. The latter indicates that electron surface scattering is completely diffuse, which is typical for rough surfaces.^44^ As the surface scattering contribution is most relevant in shallow Ag NW grids, where the surface is the roughest, it is not surprising that the fit leads to *p* = 0.

From our data and the model, we see that surface scattering is responsible for the strong increase in resistivity for Ag NWs with an aspect ratio ≲1.5, and it stabilizes to a constant value of ρ/ρ_Ag_ ≈ 1.7–1.8 for NW aspect ratios ≥2.3. This result highlights not only the high quality of our deposits (resistivity <2 times that of bulk Ag) but also the need for high aspect ratio NWs.

Now, we turn our attention to the optical properties of the Ag NW grids. The transmission of the Ag NW grids is obtained by normalizing the experimentally obtained transmission spectra of the Ag NW/ITO/glass by the experimentally obtained ITO reference spectra of each corresponding sample (for more details, see section S3 of the Supporting Information). The normalized transmission spectra for three different grid heights of the 2 μm series are shown in Figure 3b. Despite the strongly diffracting nature of the sample, no diffraction signatures are observed in the transmission spectra for all heights. This is most likely due to the fact that we use a focused noncoherent, nonpolarized light source. In general, it is found that the transmission is quite flat over a broad spectral range of 500–1200 nm, which uniformly decreases with increasing grid height. The dip in the normalized transmission spectra around 400 nm may be attributed to the increasing contribution of the surface plasmon resonance (SPR) of individual Ag NWs (for more details, see section S9 of the Supporting Information). The overall trend of decreasing transmission with increasing height is also reflected in the average transmission obtained by taking the AM1.5G spectrum weighted average of the individual transmission spectra over the spectral range 350–1200 nm (Figure S11). The decreased average transmission in taller grids is mainly the result of the increased shading of the grid due to the trapezoid shape of the wires (i.e., taller wires are wider). The average transmission is indeed very similar to that expected from geometric shading (see the inset in Figure 3b), where *T*_geo_ = (*L* – *w*)^2^/*L*^2^, with *w* and *L* being the width and pitch of the grid, respectively.

**Figure 4:**
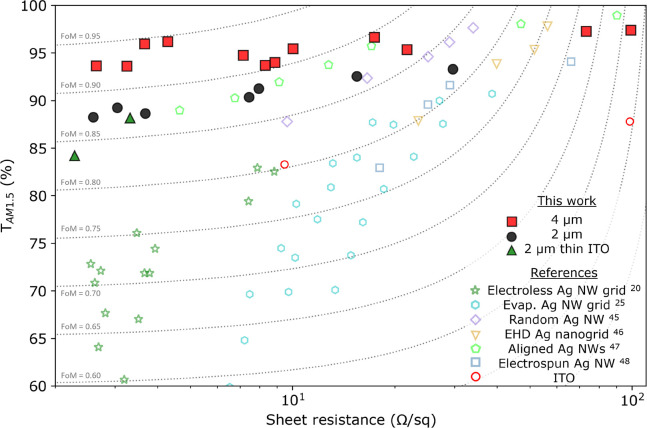
Performance characterization of the Ag NW grids, including similar systems found in the literature (electroless Ag nanogrids,^20^ evaporated Ag nanogrids,^25^ random Ag NWs,^45^ EHD printed Ag nanogrids,^46^ aligned Ag NWs,^47^ electrospun Ag NWs).^48^ The data from this work are represented by red squares, black circles, and green triangles for the 2, 4, and 2 μm on thin ITO samples, respectively. Five different values of FoM are represented by the dashed gray lines.

Finally, we compile both the optical and electronic characteristics in a typical transmittance vs sheet resistance plot, as shown in Figure 4. Here, we also included the used ITO substrate and several other Ag NW networks from the literature for reference. In all the previous works with colloidal-based NW networks, there is a clear trade-off between transparency and sheet resistance. Namely, resistivity is reduced at the expense of transparency by introducing either wider NWs or more NWs. Similarly, the thickness is the main tuning knob for adjusting sheet resistance in ITO, which has a direct impact on its transparency.

On the contrary, our approach reduces the sheet resistance through anisotropic addition of material only in the vertical direction, keeping the optical footprint (almost) untouched. Thus, the transmittance of the grids and the carrier collection are not significantly affected by decreasing the sheet resistance. While the average transmittance is tuned by the grid filling fraction (∝ *w*/*L*), the sheet resistance is independently controlled by the NW aspect ratio (*h*/*w*). As such, by tweaking the aspect ratio in the 4 μm pitch grid between ∼0.5 and 3.5, we cover about 2 orders of magnitude in sheet resistance with less than 3% absolute change in transmittance.

Comparing the performance of various TEs is not straightforward. Often, only the sheet resistance and transparency (either at 550 nm or weighted by the AM1.5G spectrum) are specified. Two commonly used figures of merit (FoM) for TEs are the Haacke FoM^49^ and the Dressel–Grüner (DG) FoM.^50^ The Haacke FoM considers the ratio of transparency (raised to a power of 10) to the sheet resistance. On the other hand, the DG FoM accounts for the electron response to electric fields, either dynamic (light) or static (voltage). While the sheet resistance is determined by the electrical DC (direct current) conductivity, the transmittance is determined by the optical conductivity. Both the Haacke and DG FoMs hold only for thin films.^51^ In grid-like networks, their value can be made arbitrarily large, as the sheet resistance decreases by increasing the pitch and width proportionally. In this case, transparency is maintained, while the sheet resistance is reduced. A more relevant FoM for comparing all kinds of TEs is defined by Anand et al., developed explicitly for assessing TE performance in photovoltaic applications.^52^ The FoM is based on the impact of sheet resistance and transmittance on the maximum attainable power according to the detailed balance limit:
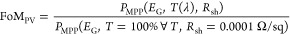
2where the bandgap *E*_G_ of the absorber is taken to be the bandgap of Si (1.14 eV). The maximum power point *P*_MPP_ can be calculated by solving the single-diode implicit Shockley equation, where the short-circuit current *I*_SC_ is calculated by considering the transmission spectra of the TE weighted by the AM1.5G solar spectrum, and the series resistance of the device is determined by the sheet resistance of the TE. More details about the calculation of the FoM can be found in section S10 of the Supporting Information.

FoM isolines are shown in Figure 4, as given by eq 2. At low sheet resistance values, the output power of the device is limited by the total amount of absorbed photon. Therefore, for a specific solar cell configuration, improving transparency is more important than decreasing the sheet resistance at those low values (<10 Ω/sq). Interestingly, most literature values exhibit a decrease in the exact FoM as the sheet resistance decreases due to a significant decrease in transmission. On the contrary, our approach decreases the sheet resistance without compromising transmittance and carrier collection by maintaining a constant distance from the exciton generated in the active layer to the Ag NW grid.

Table 1 summarizes the performance of the best electrochemically grown Ag NW grid (*w* = 99 nm, *L* = 4 μm, *h* = 345 nm) and those for other similar Ag nanowire TEs, as well as for 80 nm thick ITO, which is used as industrial standard.^6,9,25,53^ From the table, one can clearly see how the electrochemically grown Ag NW grid largely outperforms all other nanoscale approaches. This result was obtained for the most sparse grid with the largest aspect ratio. We expect that even more sparse grids (i.e., larger pitch) and even higher aspect ratios will lead to even higher FoMs. However, at some point, potential mechanical and electronic stability issues of the grids must be considered.

**Table 1:** Comparison of Different Ag NW-Based TEs Reported in the Literature

	*R*_sh_ (Ω/sq)	*T*_AM1.5G_ (%)	FoM Haacke (mΩ^–1^)	FoM DG	FoM_PV_
this work	3.7	95.9	179.9	2447	0.944
aligned Ag nanowires^47^	17.1	95.7	37.8	500	0.888
Cu microgrids^28^	0.03	86.6	7816.5	83 321	0.866
randomly dispersed Ag NWs^45^	16.6	92.4	27.3	281	0.861
evaporated Ag nanowire grids^25^	17.2	87.7	15.7	162	0.819
electrospun Ag random nanowires^48^	29.0	91.6	14.4	146	0.810
electroless filled Ag nanowire grids^20^	7.9	82.9	19.5	244	0.805
ITO (used in this work)	9.5	83.2	17.0	208	0.804
electrohydrodynamic printed Ag nanogrids^46^	23.5	87.8	11.7	120	0.799
ITO (industrial standard)^25^	58.2	87.7	4.6	48	0.683

It should be noted that Chen et al. demonstrated electrochemically grown Cu microgrids (*L* = 320 μm) using very large pitch distances that exhibit very high Haacke and DG FoMs.^28^ However, as discussed earlier, the FoMs of Haacke and DG can be made to be arbitrarily large by increasing the pitch. When looking at the PV-oriented FoM introduced by Anand et al., despite the extremely low sheet resistance of the Cu microgrid, the power output of the solar cell is still worse than that expected by our nanowire grids. Furthermore, it is important to keep in mind that in solar cells, the pitch distance will be limited to the diffusion length of the minority carrier of the underlying layer. Especially amorphous silicon, perovskite, CIGS, or organic solar cells suffer from short diffusion lengths,^5,9−11^ limiting the pitch only to tens of microns.

An additional potential advantage of NW grids with pitch distances on the order of the wavelength of light (up to a few microns) is that the grid adds in-plane momentum to the incident light that can help trap the light in thin-film solar cells. Smart geometrical designing of the NW grid can thus have dual optical–electric functionality in thin-film solar cells.

## 4 Conclusion

In this work, we demonstrated the large-area (0.95 cm^2^) fabrication of highly performing transparent silver nanowire electrodes by using a combination of substrate conformal lithography and electrochemical filling. The lift-off-free and bottom-up nature of this method allowed us to grow grids with high aspect ratio NWs, resulting in highly transparent (95.9%) and low sheet resistance (3.7 Ω/sq) TEs.

We showed that by increasing the NW height, the grid resistance decreases while the NW grid footprint remains constant without affecting carrier collection. We demonstrated that the FoM of our Ag NW grids is precisely controlled by adjusting the grid height through the deposition time, resulting in a large exact FoM that has been specified for photovoltaic applications.

While in this work we mostly focused on the NW grid transparency, the design can be taken a step further by incorporating smart optical functionalities, such as tailored diffraction and other forms of light steering. The presented method is highly scalable, and it takes advantage of the fact that nanoimprint lithography has been demonstrated in an industrial roll-to-roll process and that electroplating is a well-established industrial technology.

This approach can be generalized to the large-area nanofabrication of a wide range of metals and nanostructure designs with the potential to be used for multiple applications. The main limitation of the method is the need for a conductive substrate, such as ITO, to enable electrochemical deposition. Although grid transfer onto other substrates may be possible, we showed that the grids are equally uniform and have the same material quality when grown on substrates of higher resistance (i.e., 100 Ω/sq ITO). Consequently, we infer that uniform NW grids may be grown directly on the emitter layer of a solar cell, which will be the subject of future work.

## Supporting Information Available

Schematic of the electrochemical cell; formula for decoupling the sheet resistance of the Ag NW grid from its substrate; formula for calculating the AM1.5G average transmission; SEM images of Ag NW grids fabricated on Si substrates; large-area SEM images of the Ag NW grids; titled SEM image highlighting conformal growth; formula for calculating the NW grid height from the total transferred charge; Scherrer equation for calculating the average grain size; FDTD simulations of the Ag NW grids, validating the used normalization method; and formulas for calculating the three different FoMs (PDF)

## References

[ref1] Kim D. Y.; Kim M.-J.; Sung G.; Sun J.-Y. Stretchable and reflective displays: materials, technologies and strategies. Nano Convergence 2019, 6, 21 10.1186/s40580-019-0190-5. 31218437 PMC6584625

[ref2] Granqvist C. G. Electrochromics for smart windows: Oxide-based thin films and devices. Thin Solid Films 2014, 564, 1–38. 10.1016/j.tsf.2014.02.002.

[ref3] Oh Y. S.; Choi D. Y.; Sung H. J. Direct imprinting of thermally reduced silver nanoparticles via deformation-driven ink injection for high-performance, flexible metal grid embedded transparent conductors. RSC Adv. 2015, 5, 64661–64668. 10.1039/C5RA09431C.

[ref4] Salehi A.; Fu X.; Shin D.-H.; So F. Recent Advances in OLED Optical Design. Adv. Funct. Mater. 2019, 29, 1808803 10.1002/adfm.201808803.

[ref5] van de Groep J.; Gupta D.; Verschuuren M. A.; M Wienk M.; Janssen R. A. J.; Polman A. Large-area soft-imprinted nanowire networks as light trapping transparent conductors. Sci. Rep. 2015, 5, 11414 10.1038/srep11414. 26091006 PMC5155569

[ref6] Han C.; Santbergen R.; van Duffelen M.; Procel P.; Zhao Y.; Yang G.; Zhang X.; Zeman M.; Mazzarella L.; Isabella O. Towards bifacial silicon heterojunction solar cells with reduced TCO use. Progress in Photovoltaics: Research and Applications 2022, 30, 750–762. 10.1002/pip.3550.

[ref7] Chavan G. T.; Kim Y.; Khokhar M. Q.; Hussain S. Q.; Cho E.-C.; Yi J.; Ahmad Z.; Rosaiah P.; Jeon C.-W. A Brief Review of Transparent Conducting Oxides (TCO): The Influence of Different Deposition Techniques on the Efficiency of Solar Cells. Nanomaterials 2023, 13, 1226 10.3390/nano13071226. 37049320 PMC10096935

[ref8] Park H.; Lee Y. J.; Park J.; Kim Y.; Yi J.; Lee Y.; Kim S.; Park C. K.; Lim K. J. Front and Back TCO Research Review of a-Si/c-Si Heterojunction with Intrinsic Thin Layer (HIT) Solar Cell. Transactions on Electrical and Electronic Materials 2018, 19, 165–172. 10.1007/s42341-018-0026-8.

[ref9] Sun Z.; Chen X.; He Y.; Li J.; Wang J.; Yan H.; Zhang Y. Toward Efficiency Limits of Crystalline Silicon Solar Cells: Recent Progress in High-Efficiency Silicon Heterojunction Solar Cells. Adv. Energy Mater. 2022, 12, 2200015 10.1002/aenm.202200015.

[ref10] Lin R.; Xu J.; Wei M.; Wang Y.; Qin Z.; Liu Z.; Wu J.; Xiao K.; Chen B.; Park S. M.; Chen G.; Atapattu H. R.; Graham K. R.; Xu J.; Zhu J.; Li L.; Zhang C.; Sargent E. H.; Tan H. All-perovskite tandem solar cells with improved grain surface passivation. Nature 2022, 603, 73–78. 10.1038/s41586-021-04372-8. 35038717

[ref11] Zhu L.; Zhang M.; Xu J.; Li C.; Yan J.; Zhou G.; Zhong W.; Hao T.; Song J.; Xue X.; Zhou Z.; Zeng R.; Zhu H.; Chen C.-C.; MacKenzie R. C. I.; Zou Y.; Nelson J.; Zhang Y.; Sun Y.; Liu F. Single-junction organic solar cells with over 19% efficiency enabled by a refined double-fibril network morphology. Nat. Mater. 2022, 21, 656–663. 10.1038/s41563-022-01244-y. 35513501

[ref12] de Boer M. A.; Lammertsma K. Scarcity of Rare Earth Elements. ChemSusChem 2013, 6, 2045–2055. 10.1002/cssc.201200794. 24009098

[ref13] Holman Z. C.; Descoeudres A.; Barraud L.; Fernandez F. Z.; Seif J. P.; De Wolf S.; Ballif C. Current Losses at the Front of Silicon Heterojunction Solar Cells. IEEE Journal of Photovoltaics 2012, 2, 7–15. 10.1109/JPHOTOV.2011.2174967.

[ref14] Knight M. W.; van de Groep J.; Bronsveld P. C.; Sinke W. C.; Polman A. Soft imprinted Ag nanowire hybrid electrodes on silicon heterojunction solar cells. Nano Energy 2016, 30, 398–406. 10.1016/j.nanoen.2016.10.011.

[ref15] Qiu D.; Duan W.; Lambertz A.; Eberst A.; Bittkau K.; Rau U.; Ding K. Transparent Conductive Oxide Sputtering Damage on Contact Passivation in Silicon Heterojunction Solar Cells with Hydrogenated Nanocrystalline Silicon. Solar RRL 2022, 6, 2200651 10.1002/solr.202200651.

[ref16] Tavakoli N.; Spalding R.; Lambertz A.; Koppejan P.; Gkantzounis G.; Wan C.; Röhrich R.; Kontoleta E.; Koenderink A. F.; Sapienza R.; Florescu M.; Alarcon-Llado E. Over 65% Sunlight Absorption in a 1 μm Si Slab with Hyperuniform Texture. ACS Photonics 2022, 9, 1206–1217. 10.1021/acsphotonics.1c01668. 35480493 PMC9026274

[ref17] Atwater H. A.; Polman A. Plasmonics for improved photovoltaic devices. Nat. Mater. 2010, 9, 205–213. 10.1038/nmat2629. 20168344

[ref18] Garnett E. C.; Ehrler B.; Polman A.; Alarcon-Llado E. Photonics for Photovoltaics: Advances and Opportunities. ACS Photonics 2021, 8, 61–70. 10.1021/acsphotonics.0c01045. 33506072 PMC7821300

[ref19] Neder V.; Tabernig S. W.; Polman A. Detailed-balance efficiency limits of two-terminal perovskite/silicon tandem solar cells with planar and Lambertian spectral splitters. Journal of Photonics for Energy 2022, 12, 015502 10.1117/1.JPE.12.015502.

[ref20] Sciacca B.; van de Groep J.; Polman A.; Garnett E. C. Solution-Grown Silver Nanowire Ordered Arrays as Transparent Electrodes. Adv. Mater. 2016, 28, 905–909. 10.1002/adma.201504045. 26632271

[ref21] Gao J.; Kempa K.; Giersig M.; Akinoglu E. M.; Han B.; Li R. Physics of transparent conductors. Adv. Phys. 2016, 65, 553–617. 10.1080/00018732.2016.1226804.

[ref22] Guo H.; Lin N.; Chen Y.; Wang Z.; Xie Q.; Zheng T.; Gao N.; Li S.; Kang J.; Cai D.; Peng D. L. Copper nanowires as fully transparent conductive electrodes. Sci. Rep. 2013, 3, 2323 10.1038/srep02323. 23900572 PMC3728602

[ref23] Zhu Y.; Deng Y.; Yi P.; Peng L.; Lai X.; Lin Z. Flexible Transparent Electrodes Based on Silver Nanowires: Material Synthesis, Fabrication, Performance, and Applications. Advanced Materials Technologies 2019, 4, 1900413 10.1002/admt.201900413.

[ref24] Kumar A.; Kumar M.; Goyat M. S.; Avasthi D. K. A review of the latest developments in the production and applications of Ag-nanowires as transparent electrodes. Materials Today Communications 2022, 33, 104433 10.1016/j.mtcomm.2022.104433.

[ref25] Van De Groep J.; Spinelli P.; Polman A. Transparent conducting silver nanowire networks. Nano Lett. 2012, 12, 3138–3144. 10.1021/nl301045a. 22554260

[ref26] Catrysse P. B.; Fan S. Nanopatterned metallic films for use as transparent conductive electrodes in optoelectronic devices. Nano Lett. 2010, 10, 2944–2949. 10.1021/nl1011239. 20698607

[ref27] Khan A.; Liang C.; Huang Y.-T.; Zhang C.; Cai J.; Feng S.-P.; Li W.-D. Template-Electrodeposited and Imprint-Transferred Microscale Metal-Mesh Transparent Electrodes for Flexible and Stretchable Electronics. Adv. Eng. Mater. 2019, 21, 1900723 10.1002/adem.201900723.

[ref28] Chen X.; Nie S.; Guo W.; Fei F.; Su W.; Gu W.; Cui Z. Printable High-Aspect Ratio and High-Resolution Cu Grid Flexible Transparent Conductive Film with Figure of Merit over 80 000. Advanced Electronic Materials 2019, 5, 1800991 10.1002/aelm.201800991.

[ref29] Zhang H.; Zhang N.; Gilchrist M.; Fang F. Advances in precision micro/nano-electroforming: A state-of-the-art review. Journal of Micromechanics and Microengineering 2020, 30, 103002 10.1088/1361-6439/aba017.

[ref30] Verschuuren M. A.; Megens M.; Ni Y.; Van Sprang H.; Polman A. Large area nanoimprint by substrate conformal imprint lithography (SCIL). Advanced Optical Technologies 2017, 6, 243–264. 10.1515/aot-2017-0022.

[ref31] Khan A.; Lee S.; Jang T.; Xiong Z.; Zhang C.; Tang J.; Guo L. J.; Li W.-D. High-Performance Flexible Transparent Electrode with an Embedded Metal Mesh Fabricated by Cost-Effective Solution Process. Small 2016, 12, 3021–3030. 10.1002/smll.201600309. 27027390

[ref32] Sandmann G.; Dietz H.; Plieth W. Preparation of silver nanoparticles on ITO surfaces by a double-pulse method. J. Electroanal. Chem. 2000, 491, 78–86. 10.1016/S0022-0728(00)00301-6.

[ref33] Bleiji Y.; Dieperink M.; Schuringa I.; Sun H.; Alarcon-Llado E. Influence of the crystallographic texture of ITO on the electrodeposition of silver nanoparticles. RSC Adv. 2023, 13, 6490–6497. 10.1039/D3RA00577A. 36845599 PMC9949353

[ref34] Fuchs K. The conductivity of thin metallic films according to the electron theory of metals. Mathematical Proceedings of the Cambridge Philosophical Society 1938, 34, 100–108. 10.1017/S0305004100019952.

[ref35] Sondheimer E. The mean free path of electrons in metals. Adv. Phys. 1952, 1, 1–42. 10.1080/00018735200101151.

[ref36] Mayadas A. F.; Shatzkes M. Electrical-Resistivity Model for Polycrystalline Films: the Case of Arbitrary Reflection at External Surfaces. Phys. Rev. B 1970, 1, 1382 10.1103/PhysRevB.1.1382.

[ref37] Steinhögl W.; Schindler G.; Steinlesberger G.; Engelhardt M. Size-dependent resistivity of metallic wires in the mesoscopic range. Phys. Rev. B 2002, 66, 075414 10.1103/PhysRevB.66.075414.

[ref38] Steinhögl W.; Schindler G.; Steinlesberger G.; Traving M.; Engelhardt M. Comprehensive study of the resistivity of copper wires with lateral dimensions of 100 nm and smaller. J. Appl. Phys. 2005, 97, 023706 10.1063/1.1834982.

[ref39] Kanter H. Slow-Electron Mean Free Paths in Aluminum, Silver, and Gold. Phys. Rev. B 1970, 1, 522–536. 10.1103/PhysRevB.1.522.

[ref40] Gall D. Electron mean free path in elemental metals. J. Appl. Phys. 2016, 119, 085101 10.1063/1.4942216.

[ref41] Xiang C.; Kung S. C.; Taggart D. K.; Yang F.; Thompson M. A.; Güell A. G.; Yang Y.; Penner R. M. Lithographically patterned nanowire electrodeposition: A method for patterning electrically continuous metal nanowires on dielectrics. ACS Nano 2008, 2, 1939–1949. 10.1021/nn800394k. 19206435

[ref42] Kung S. C.; Xing W.; Donavan K. C.; Yang F.; Penner R. M. Photolithographically patterned silver nanowire electrodeposition. Electrochim. Acta 2010, 55, 8074–8080. 10.1016/j.electacta.2010.02.075.

[ref43] Patterson A. L. The Scherrer Formula for X-Ray Particle Size Determination. Phys. Rev. 1939, 56, 978–982. 10.1103/PhysRev.56.978.

[ref44] Gall D. The search for the most conductive metal for narrow interconnect lines. J. Appl. Phys. 2020, 127, 050901 10.1063/1.5133671.

[ref45] Leem D.-S.; Edwards A.; Faist M.; Nelson J.; Bradley D. D. C.; de Mello J. C. Efficient Organic Solar Cells with Solution-Processed Silver Nanowire Electrodes. Adv. Mater. 2011, 23, 4371–4375. 10.1002/adma.201100871. 21861269

[ref46] Schneider J.; Rohner P.; Thureja D.; Schmid M.; Galliker P.; Poulikakos D. Electrohydrodynamic NanoDrip Printing of High Aspect Ratio Metal Grid Transparent Electrodes. Adv. Funct. Mater. 2016, 26, 833–840. 10.1002/adfm.201503705.

[ref47] Feng X.; Wang L.; Huang Y. Y. S.; Luo Y.; Ba J.; Shi H. H.; Pei Y.; Zhang S.; Zhang Z.; Jia X.; Lu B. Cost-Effective Fabrication of Uniformly Aligned Silver Nanowire Microgrid-Based Transparent Electrodes with Higher than 99% Transmittance. ACS Appl. Mater. Interfaces 2022, 14, 39199–39210. 10.1021/acsami.2c09672. 35976981

[ref48] Luo Y.; Ning T.; Pei Y.; Feng X.; Zhang S.; Lu B.; Wang L. High-performance and tailored honeycombed Ag nanowire networks fabricated by a novel electrospray assisted etching process. Appl. Surf. Sci. 2022, 571, 151081 10.1016/j.apsusc.2021.151081.

[ref49] Haacke G. New figure of merit for transparent conductors. J. Appl. Phys. 1976, 47, 4086–4089. 10.1063/1.323240.

[ref50] Dressel M.; Grüner G. In Electrodynamics of Solids: Optical Properties of Electrons in Matter; Cambridge University Press, 2002.

[ref51] De S.; Coleman J. N. Are There Fundamental Limitations on the Sheet Resistance and Transmittance of Thin Graphene Films?. ACS Nano 2010, 4, 2713–2720. 10.1021/nn100343f. 20384321

[ref52] Anand A.; Islam M. M.; Meitzner R.; Schubert U. S.; Hoppe H. Introduction of a Novel Figure of Merit for the Assessment of Transparent Conductive Electrodes in Photovoltaics: Exact and Approximate Form. Adv. Energy Mater. 2021, 11, 2100875 10.1002/aenm.202100875.

[ref53] Caudevilla D.; García-Hemme E.; San Andrés E.; Pérez-Zenteno F.; Torres I.; Barrio R.; García-Hernansanz R.; Algaidy S.; Olea J.; Pastor D.; del Prado A. Indium tin oxide obtained by high pressure sputtering for emerging selective contacts in photovoltaic cells. Materials Science in Semiconductor Processing 2022, 137, 106189 10.1016/j.mssp.2021.106189.

