# Supplementary material for: High Aspect Ratio Silver Nanogrids by Bottom-Up Electrochemical Growth as Transparent Electrode
Source: ACS Appl Opt Mater. 2024 Mar 13;2(3):508–16. doi: 10.1021/acsaom.4c00037 (PMC10964241; doi:10.1021/acsaom.4c00037)
Supplement: Supplementary file 1 — ot4c00037_si_001.pdf [file ot4c00037_si_001.pdf]

Supporting Information: High Aspect Ratio Silver Nanogrids by Bottom-Up Electrochemical Growth as Transparent Electrode

*Yorick Bleiji, Andrea Cordaro, Stefan. W. Tabernig and Esther Alarcon-Llado**

Center for Nanophotonics, AMOLF, Science Park 104, 1098 XG, Amsterdam, The Netherlands

*Corresponding Author:
E-mail address: e.alarconllado@amolf.nl

S1 Electrochemical cell

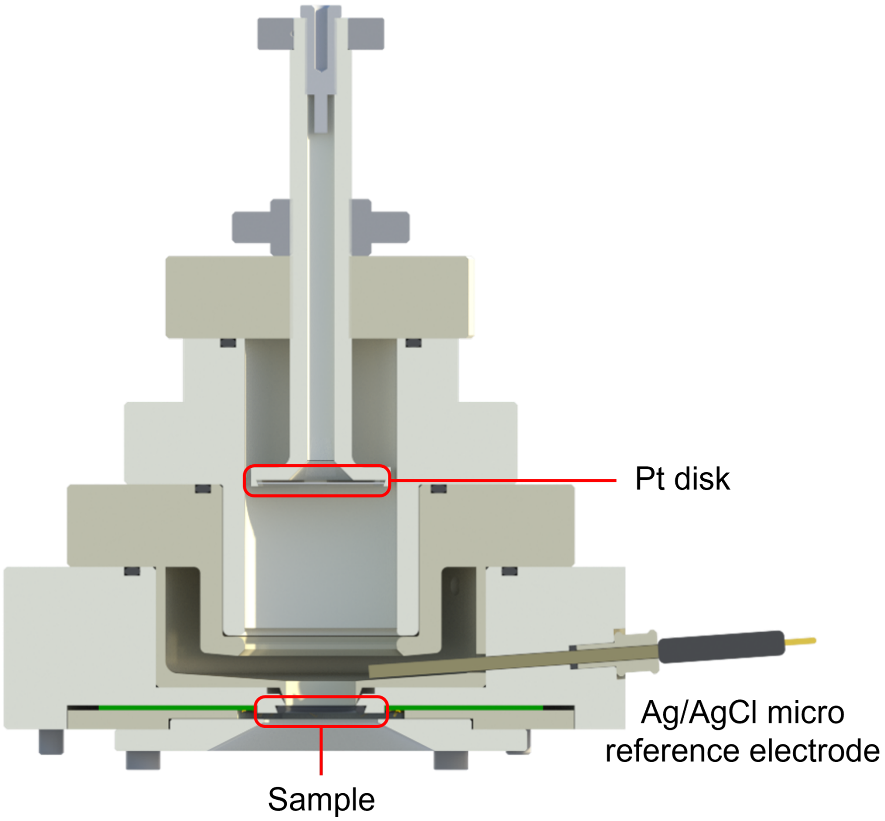

Figure S1: Schematic of the used electrochemical cell for the superfilling of Ag. The distance between the CE and the WE is adjustable and was set to 15 mm. The volume of the used electrolyte is 24 mL.

S2 Obtaining resistivity by the decoupling the sheet resistance

The sheet resistance R_{sh}^{Ag} of the samples was obtained directly before and directly after the electrochemical deposition of silver, using the van der Pauw method. Four gold pins located at the bottom of the electrochemical cell, as shown in Figure S2(a), were used to perform a 4-point probe resistance measurement using a SP-300 Bio-Logic potentiostat. A clover-leaf type of configuration was used, where four scratches were made on the ITO substrate to make sure that the current runs through the center region of the sample. A representative sample after the filling of the trenches including the 4 scratches into the ITO substrate is shown in Figure S2(b). The sheet resistance was calculated using the van der Pauw formula:

$$e^{-\pi R_{hor}/R_{sh}} + e^{-\pi R_{ver}/R_{sh}} = 1 \tag{S1}$$

By assuming that the ITO substrate and the Ag NW grids are two parallel resistors, the sheet resistance of the bare Ag NW grids could be calculated by:

$$R_{sh}^{Ag} = \left(\frac{1}{R_{sh}^{ITO+Ag}} - \frac{1}{R_{sh}^{ITO}} \right)^{-1} \tag{S2}$$

The resistivity ρ of the wires was extracted from the sheet resistance using an effective height as if the grids were a film:

$$R_{sh} = \frac{\rho}{h_{eff}} \tag{S3}$$

where $h_{eff} = {}^{hw}/_{L}$, where h is the height of the Ag NW grids, w the average width of the trapezoidal shaped cross section, and L is the pitch.

Another method to extract the resistivity is by using Kirchoff's rules, where the total sheet resistance is the sum of the individual wires in the horizontal direction (resistors in series), normalized by the wires in the vertical directions (resistors in parallel).[1] The sheet resistance R_{sh} for a NxN network is then given by:

$$R_{sh} = \frac{N}{N+1} R_{wire} = \frac{N}{N+1} \frac{\rho L}{wh} \tag{S4}$$

For large N, this expression is identical to the effective height method.

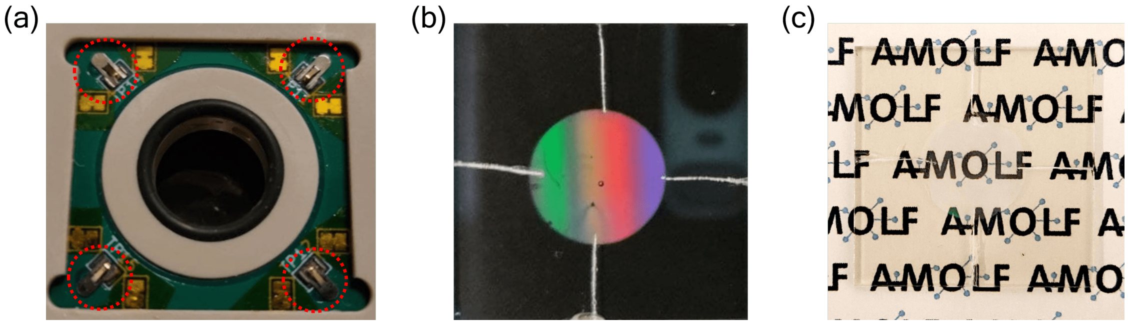

Figure S2: a) Bottom of the electrochemical cell showing the O-ring (D=11 mm) and the 4 gold pins (highlighted by the dashed red circle) used for the van der Pauw measurements. Photograph taken under b) a slight angle and c) normal incidence of a representative sample after the filling of the trenches including the scratches in the ITO.

S3 Normalized Transmission spectra

The transmission spectra of bare ITO/glass were obtained at an area of the sample where no mask and Ag NWs were present, while the transmission spectra of the Ag NW/ITO/glass were obtained in the center of the sample. The transmission spectra of purely the Ag NW grids were obtained by dividing the Ag NW/ITO/glass spectra by the ITO/glass reference spectra. The average transmission was obtained by taking an weighted average using the AM1.5G spectrum $I_s(\lambda)$ given by:

$$T_{AM1.5G} = \frac{\int_{350nm}^{1200nm} T(\lambda) I_s(\lambda) d\lambda}{\int_{350nm}^{1200nm} I_s(\lambda) d\lambda} \tag{S5}$$

The AM1.5G weighted averaged was taken over the spectral range of 350-1200 nm to exclude the strong absorption of the ITO/glass substrate (up to the dashed vertical line indicated in Figure S9).

S4 Fabrication of Ag NW grids on n-Si

To demonstrate that Ag NW grids can also be grown on other common substrates, Ag NW grids were also fabricated on heavily doped n-Si (0.008-0.02 Ω-cm). An SEM image of the fabricated Ag NW grids on n-Si in shown in Figure S3. The fabrication of the Ag NW grids on Si follows the same procedure as on ITO, with the addition of an HF vapor etch (300 s, 125 mbar, N2: 1200 sccm, EtOH: 350 sccm, HF: 300 sccm) after the O_2 reactive ion etch step.

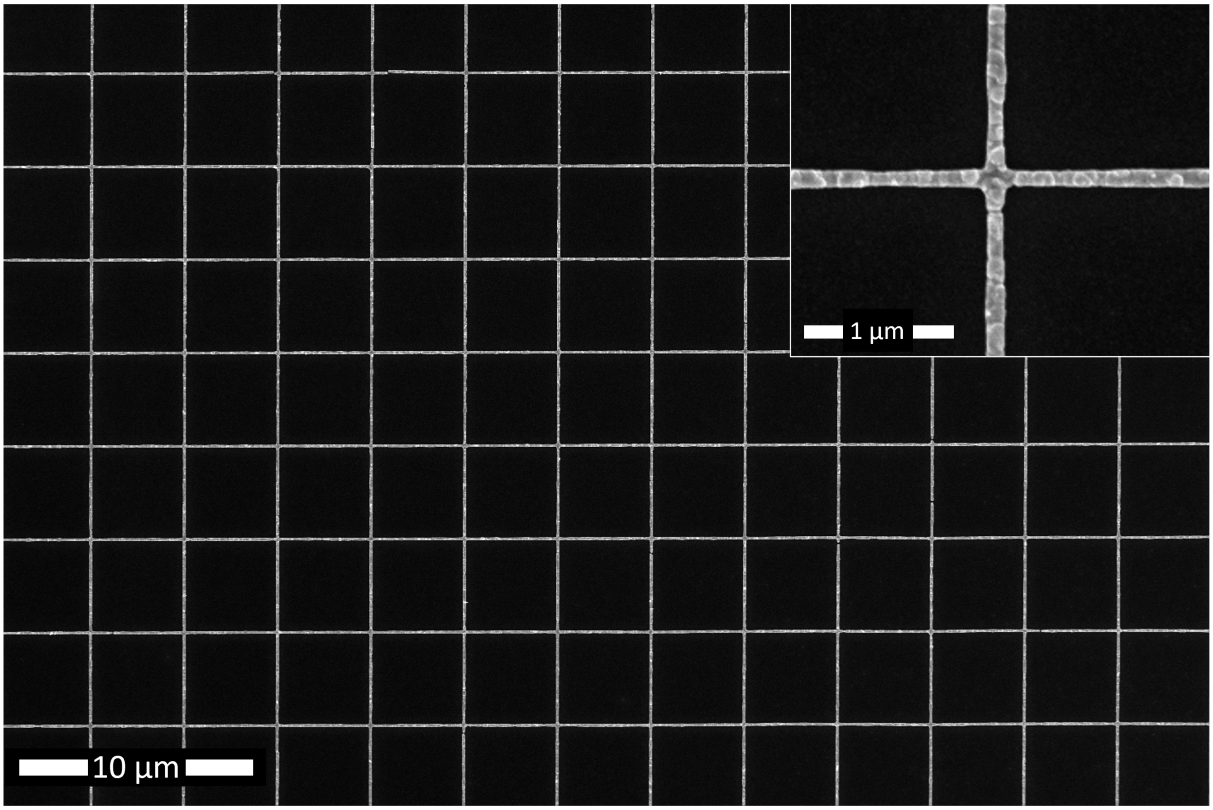

Figure S3: SEM image of a Ag NW grid grown on a n-Si substrate having a pitch of 4 μm. The inset shows the crossing of the NWs in more detail.

S5 Large area deposition

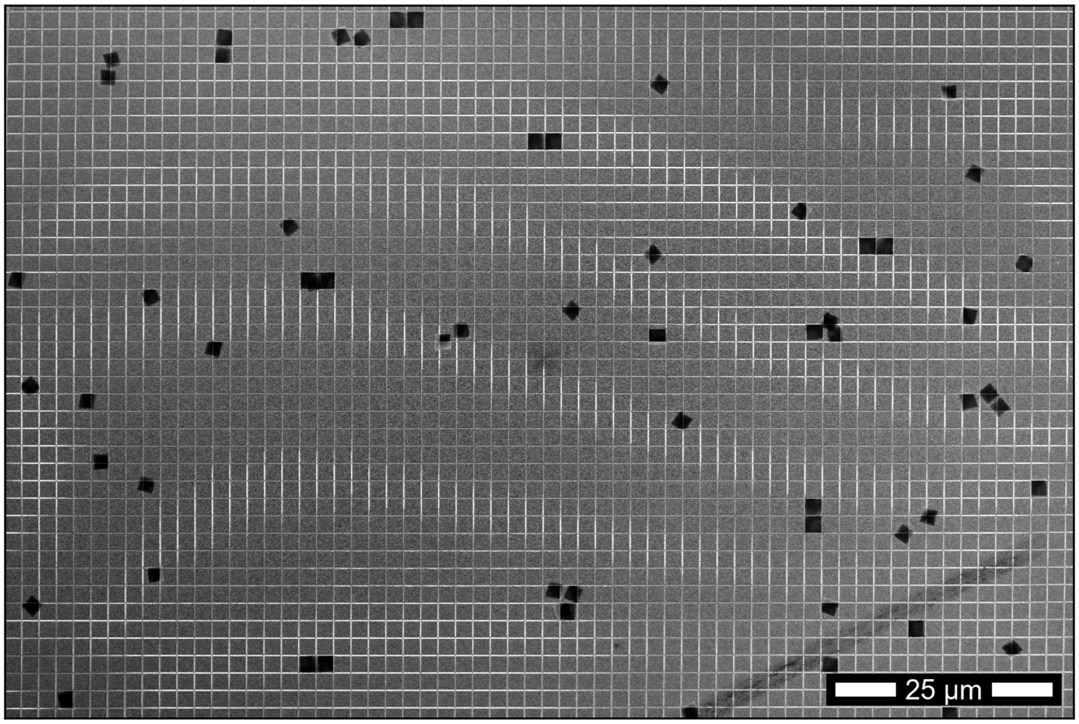

Figure S4: Large area SEM image of a sample having a pitch of 2 μm and a height of 219 nm. The black spots on the sample are sol-gel slabs, which were redeposited during the removal of the template. The wrinkles in the contrast are Moire patterns due to the overlap of the grid with the pixels of the SEM detector.

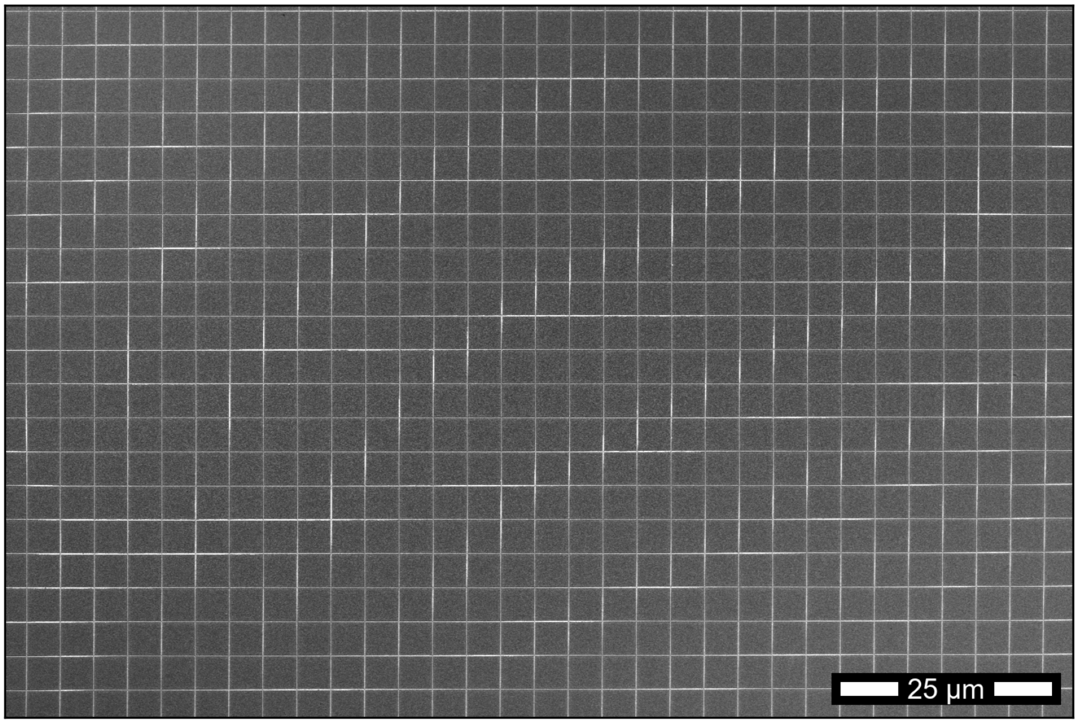

Figure S5: Large area SEM image of a sample having a pitch of 4 μm and a height of 82 nm. In this area on the sample, all the PMMA/sol-gel slabs have been successfully removed. The wrinkles in contrast are Moire patterns due to the overlap of the grid with the pixels of the SEM detector.

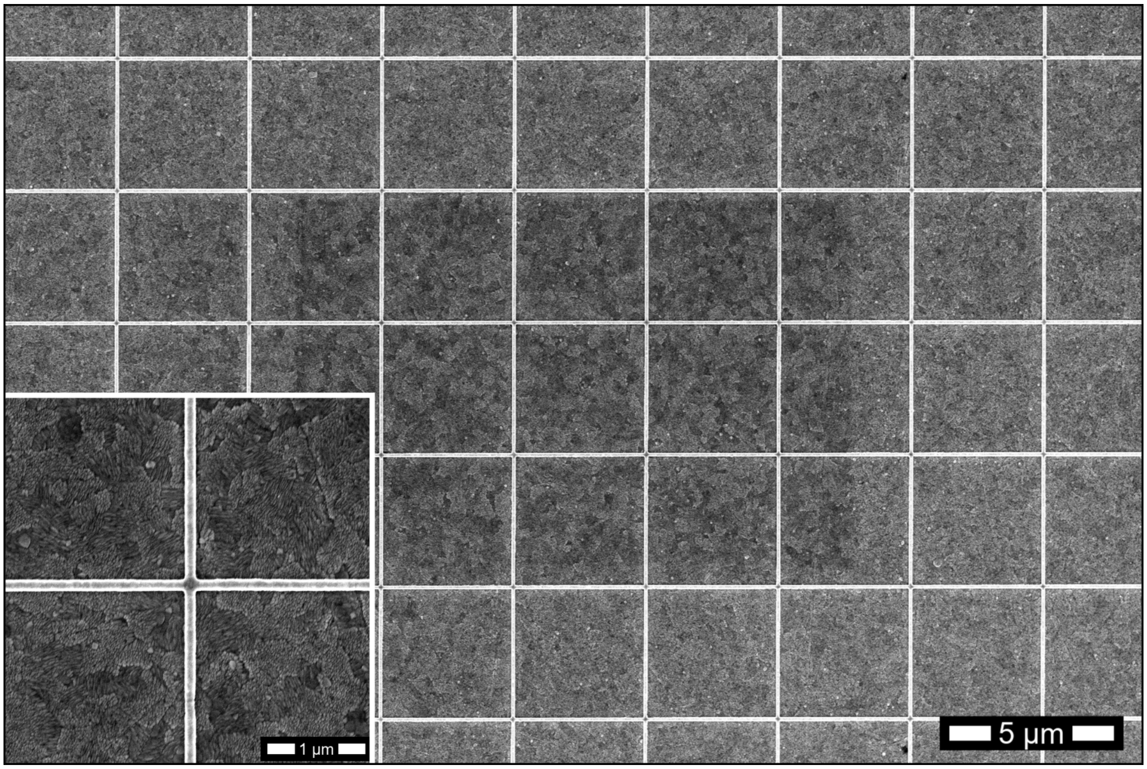

Figure S6: SEM image of a typical template-assisted electrodeposited Ag NW grid having a pitch of 4 μm. The inset shows the crossing of two NWs in more detail.

S6 Conformal growth

Figure S7 shows a false-colored SEM image of an overgrown sample with pitch 4 μm. In this false-colored image, Ag, ITO, and sol-gel are represented in gray, yellow, and blue, respectively. From this tilted SEM image, the conformal growth is clearly demonstrated. The horizontal wire shows three distinct regions. The first is a smooth wall whose smoothness is defined by the smoothness of the PMMA. On top of that, a darker region is observed, which is an indent in the Ag NW. This indent is caused by the sol-gel layer, which has a slightly smaller trench width than the PMMA layer. Above the indent, the Ag NW start to grow out of the trench, which results in a dome-like shaped deposit (unrestricted growth).

The reason behind this particular shape of the wire is represented in Figure S8. The non-isotropic reactive oxygen ion etch results in a slight undercut, meaning that the sol-gel layer is slightly extending further into the trench than the PMMA layer. The electrochemical filling of the trenches is a conformal process, so the deposit follows the shape of the template. After filling, the template is removed by dissolving the PMMA layer in acetone. However, due to the shape of the template, the sol-gel layer is trapped in the Ag NW and therefore it is hard to remove the sol-gel. After the sample was taken out the acetone, the sol-gel layer collapses and redeposits onto the ITO substrate. The suggested indent in the Ag NW is confirmed by the cross-sectional false-colored SEM image shown in Figure S8(d)

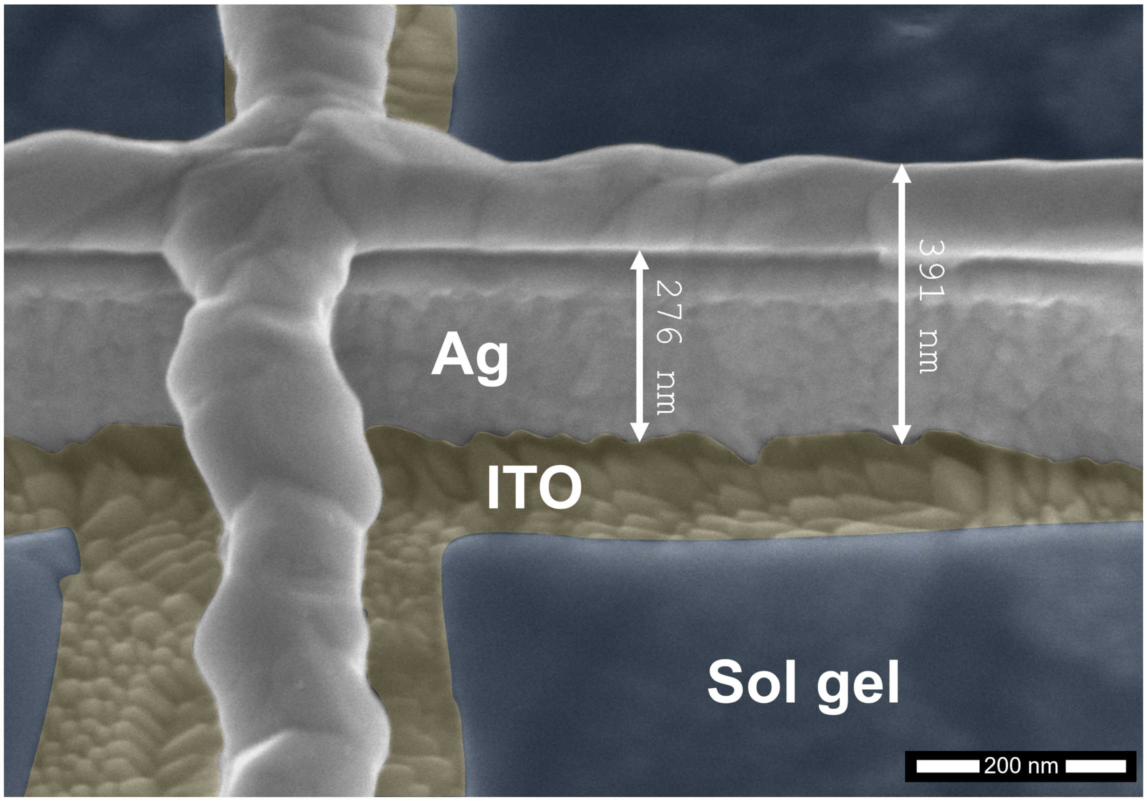

Figure S7: False-colored cross sectional SEM image of a sample with pitch 4 μm, taking with an image tilt of 45°. The Ag, ITO and sol-gel are represented in gray, yellow and blue, respectively.

S7 Extracting the grid height using the total transferred charge method

The total deposited charge Q is obtained by integrating the current over the deposition time, hereby assuming a Faradaic efficiency of 100%. Using the total footprint area of the Ag NW grids A_{grids} (homogeneous filling), the Faraday constant F, the amount of electrons involved $n = 1$, molar weight MW_{Ag} and density of silver ρ_{Ag}, the height from the transferred charge can be calculated by:

$$h_Q = \frac{Q}{nFA_{grids}} \frac{MW_{Ag}}{\rho_{Ag}} \quad , \tag{S6}$$

where

$$A_{grids} = \frac{2Lw_{ave} - w_{ave}^2}{L^2} A_{EC} \quad , \tag{S7}$$

where $A_{EC} = 0.95$ cm^2 is the electrochemical active area, w_{ave} and L are the *average* width and pitch of the Ag NW grid, respectively. Since the cross sectional of the Ag NW is not a perfect rectangle, the average width of the Ag NW was used to obtain the total footprint area of the Ag NW grids A_{grids}. The average width is obtained by taking the average of the width as seen from the top by SEM and the width at the bottom of the trench (found by extrapolating to zero time in Figure 2(a)). We therefore implicitly assume a trapezoidal shape of the wires in the calculation of the height using the total transferred charge.

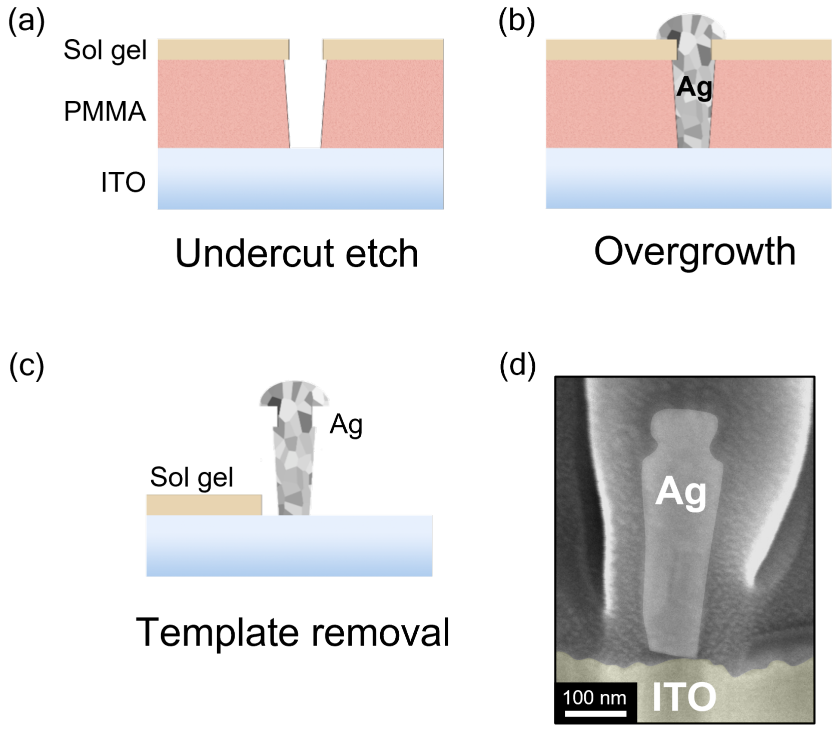

Figure S8: Schematic illustrating the conformal growth. a) The non-isotropic reactive ion etch results into an undercut type of etch. b) The electrodepostion of Ag is conformal until overgrowth. c) The template has been removed by dissolving the PMMA in acetone. Due to the encapsulation of the sol-gel by Ag NW, the sol-gel layer could not by fully removed and falls down to the substrate. d) False-colored cross section SEM image of a sample that was overgrown confirming the suggested cross-sectional shape.

S8 Average Grain Diameter

The average grain diameter was obtained by XRD using thin ITO samples (100 Ω/sq). The XRD scans were corrected by subtracting the background and removing the $K_{\alpha,2}$ peak. The average grain diameter was determined by the Scherrer equation:[2–4]

$$L = \frac{K\lambda}{\beta\cos(\theta)} \tag{S8}$$

where L is the average grain diameter, $K = 0.9$ is the Scherrer constant, λ is the wavelength of the X-ray ($\lambda = 0.154$ nm for Cu K_α), β is the FHHM peak in radians, and θ is the Bragg angle (half of the 2θ peak position). The FHHM β of Ag(111) ($2\theta = 38.4°$) was corrected for the instrument broadening by using a Corundum reference sample using the peak at $2\theta = 37.8°$.

S9 FDTD simulations

To further investigate the physical phenomena of the measured transmission spectra, 2D finite difference time domain (FDTD) simulations were performed using Lumerical by Ansys.[5] In the 2D FDTD simulations, the Ag NWs have a width and height as extracted from SEM and AFM measurements, and are either placed on 180 nm thick ITO with a semi-infinite glass substrate or surrounded by air. Periodic boundary conditions were used to simulate an infinite network with an

x-span of 2 or 4 μm. A broad band (280-1200 nm) plane wave is used at normal incidence, using two polarizations of the E-field. The final reported (non-polarized) simulated transmission spectrum is the average of the transmission spectrum for the E-field polarized along the Ag wire, and the E-field polarized perpendicular to the Ag wire. The transmission spectrum through the Ag NW/ITO/glass stack was recorded by placing a power meter 1.22 μm below the ITO/glass interface and 100 nm above the end of the simulation box. On the x-boundaries (perpendicular to the propagation of light), perfectly matched layer (PML) boundary conditions with a steep angle profile were used to prevent any unwanted backscattering at the edge of the simulation box. Optical constants for Ag and glass were obtained from Palik,[6] and the optical constant of ITO was obtained from König.[7] In the FDTD simulation, the semi-infinite glass does not include any absorption, as the k-values were set to zero. All simulated FDTD transmission spectra are corrected for by the absorption of 1.1 mm thick BK7 glass.

Figure S9 shows the experimental (red) and simulated (black) transmission spectra of a 41 nm tall and 86 nm wide Ag NW grating. The spectra overlap quite well in the spectral range of 280 to 750 nm. For longer wavelengths, however, the measured spectrum shows a consistent lower transmission, which is explained by the difference in the optical constants between the experimentally used ITO and the theoretical values used in the FDTD simulations. The broad band thin-film interference peaks around 390, 460 and 620 nm in the simulated spectrum are overlapping with the experimentally obtained spectrum, validating the ITO thickness of 180 nm used in the FDTD simulations. The sharp resonances appearing in the simulated spectrum at 400, 432, 476, 530, 608 and 728 nm are attributed to diffraction. Remarkably, these sharp diffraction signatures are not observed experimentally, which is the result of the used focused non-coherent, non-polarized light, resulting in a broadening of the peaks.

Figure S10(a) shows the simulated (solid black) and experimental (solid red) *normalized* transmission of a 41 nm tall and 86 nm wide Ag NW grating. The transmission was normalized by

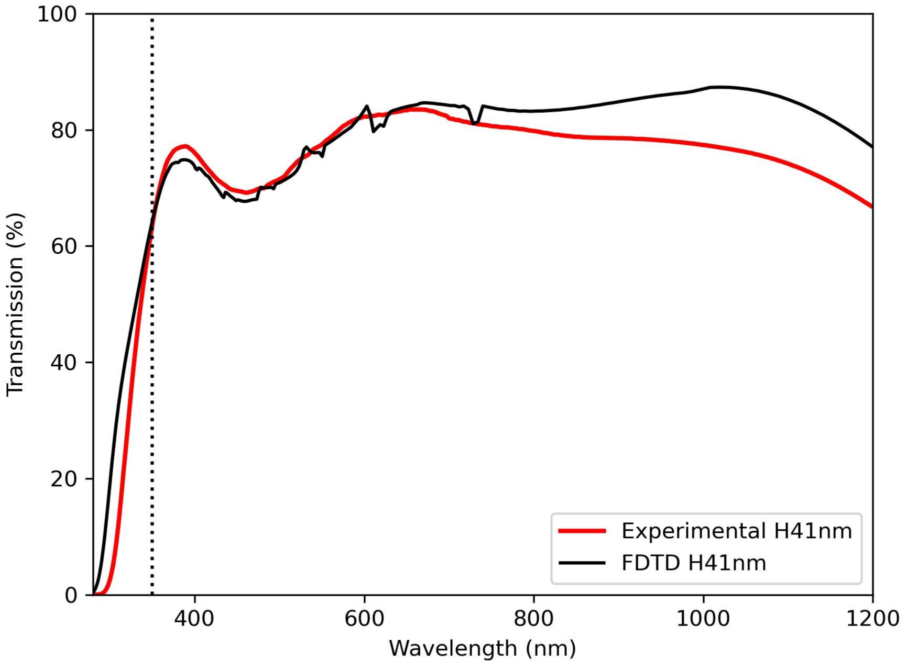

Figure S9: Simulated transmission spectra (black) of infinite Ag NW 1D grating, having a height and width of 41 nm and 86 nm, respectively, overlapped with the experimentally obtained transmission spectra (red) of Ag NW/ITO/glass. The AM1.5G weighted average was taking in the spectral range of 350-1200 nm as indicated by the dashed vertical line.

their corresponding ITO/glass transmission (e.g. simulated transmission of 180 nm thick ITO on semi-infinite glass and experimentally obtained transmission of ITO/glass). To validate this normalization method, the transmission spectrum of the same Ag NW grating surrounded by air (dashed green) is also included. Remarkably, the surface plasmon resonance (SPR) occurring around 400 nm in the transmission spectra of the Ag NW grating in air is not clearly visible in the ITO/glass normalized simulated transmission spectrum. This is due to the presence of the ITO substrate, which modifies the electric field along the edges of the Ag NW, thereby blue shifting and dampening the SPR. Furthermore, the diffraction peaks are much more pronounced when the ITO/glass substrate is present compared to the transmission in air. However, with increasing Ag NW height, the SPR becomes more prominent in both the normalized simulated as well as in the experimentally obtained normalized transmissions, as is shown in Figure S10(b) for a 288 nm tall and 115 nm wide Ag NW grating. While the diffraction peaks become more prominent in the normalized simulated transmission spectra for taller Ag NW gratings on ITO/glass and in air, the diffraction peaks do not appear in the experimentally obtained normalized transmission.

On average, the *normalized* simulated transmission on ITO/glass and in air agrees quite well with each other, which is summarized in Figure S11. In this graph, the AM1.5G weighted average of the normalized simulated transmission on ITO/glass and in air are represented by open red and black squares, respectively, for both the samples having a pitch of 2 μm and 4 μm (figures S11(a) and S11(b), respectively). The AM1.5G weighted average transmission of the two cases overlap almost perfectly, thus validating the used normalization method. The difference between the experimental average normalized transmission (solid red circles) and the FDTD simulation on ITO/glass (open black squares) is the result of different optical constants used for ITO in the simulation, as explained earlier. The same reasoning can be used for the difference between the simulated and experimental obtained average transmission of the full Ag NW/ITO/glass stack. Interestingly, the average of the experimental normalized transmission is very similar to the transmission expected from geometrical shading (based on the width and pitch of the Ag NW grating), which is summarized and discussed in the inset of Figure 3(b) in the manuscript.

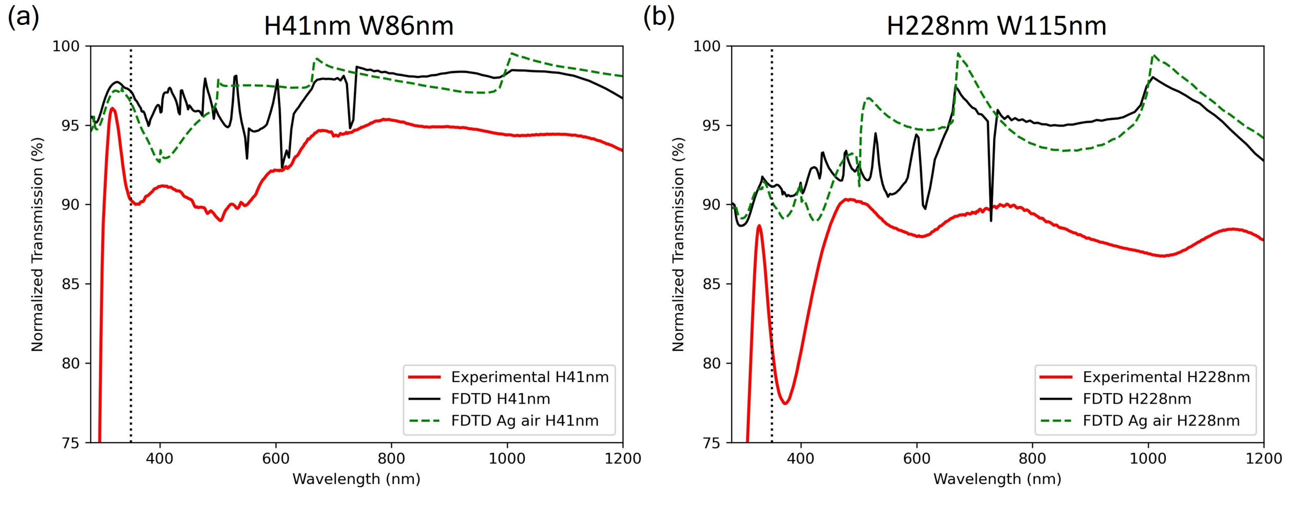

Figure S10: Normalized experimental (red) and simulated (black) transmission spectra together with the simulated transmission spectra of the same Ag NW grating in air (dashed green), for a (a) 41 nm and (b) 228 nm tall Ag NW grating.

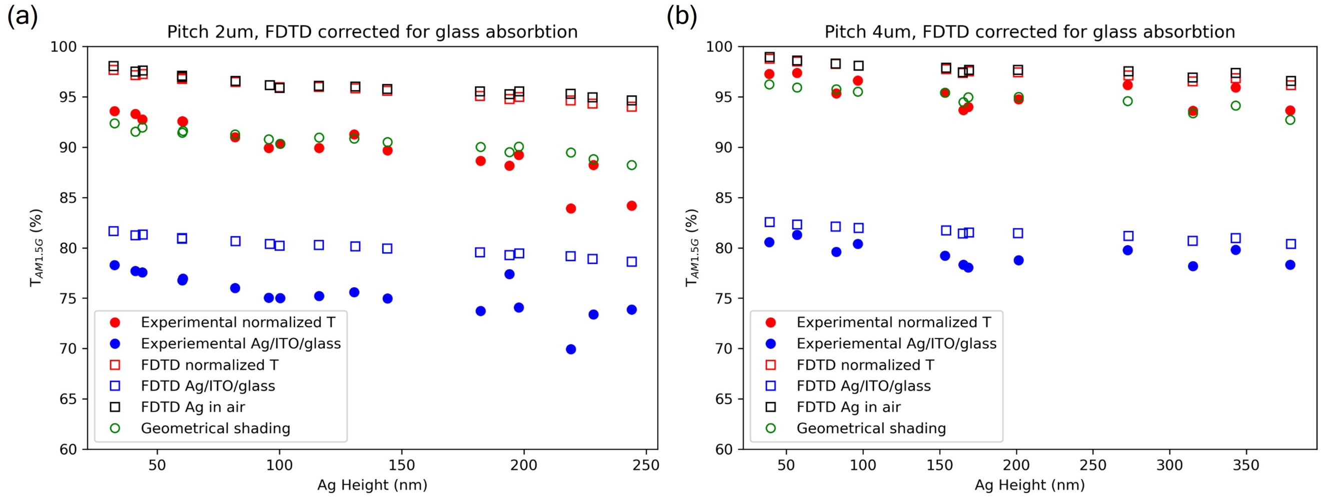

Figure S11: AM1.5G weighted average transmission as function of the Ag NW grating height, for samples having a pitch of (a) 2 μm and (b) 4 μm. Experimental and simulated data is represented by closed circles and open squares, respectively. The average transmission of the normalized spectra and total transmission of the Ag NW/ITO/glass stack are represented by red and blue, respectively. The simulated transmission of the Ag NW grating in air is represented by open black squares. The transmission expected from geometrical shading is represented by the open green circles.

S10 Figure of Merit

Two commonly used Figures of Merit (FoM) for TEs are the Haacke FoM[8] and the Dressel-Grüner (DG) FoM.[9] The FoM introduced by Haacke is given by:[8]

$$FoM_{Haacke} = \frac{T^{10}}{R_{sh}} \tag{S9}$$

where R_{sh} is the sheet resistance, and T is the average transmission, weighted by either the AM1.5G solar spectrum or the transmittance at 550 nm.

The FoM introduced by Dressel and Grüner is given by:[9]

$$FoM = \frac{\sigma_{dc}}{\sigma_{opt}} = \frac{188.5}{R_{sh}(T^{-1/2} - 1)} \tag{S10}$$

where σ_{dc} and σ_{opt} are the DC electrical and optical conductivity, respectively. As discussed in the manuscript, for metal grids these FoM can be made arbitrarily large as the sheet resistance decreases by increasing both the pitch and width proportionally, thus maintaining transparency while reducing sheet resistance.

Therefore, when comparing electrochemically grown Ag NW grids with other Ag NW-based TEs, we use the FoM as defined byAnand et al., developed explicitly to assess the performance of TE in photovoltaic applications.[10] This FoM is based on the impact of sheet resistance and transmittance spectra on the maximum attainable power according to the detailed balance limit:

$$FoM = \frac{P_{MPP}(E_G, T(\lambda), R_{sh})}{P_{MPP}(E_G, T = 100\% \forall T, R_{sh} = 0.0001\Omega/sq)} \tag{S11}$$

where the bandgap E_G of the absorber is taken to be the bandgap of Si (1.14 eV). The maximum power point $P_{MPP} = \max(I \cdot V)$ can be calculated by solving the self-consistent 1-diode implicit

Shockley equation given by:

$$I = I_0 \left[\exp \left(\frac{q(V - IR_s)}{k_B T} - 1 \right) \right] - I_{SC} \tag{S12}$$

where q is the elementary charge, k_B is the Boltzmann constant, and T is the ambient temperature. The series resistance R_s can be calculated from the sheet resistance of the TE:

$$R_s = R_{sh} \frac{l_{cell}}{3w_{cell}} \tag{S13}$$

where l_{cell} and w_{cell} are the length and width of the solar cell, respectively. We use the same parameters as Anand et al. for the calculation of the series resistance R_S, which are $l_{cell} = 5$ mm and $w_{cell} = 10$ cm.

The short circuit current I_{SC} and dark current I_0 can be calculated by the short circuit J_{SC} and dark current density J_0, multiplied by the cell area $A = l_{cell} w_{cell}$. The short circuit and dark current density both depends on the transmission spectrum of the TE. The short circuit current is given by:

$$I_{SC} = J_{SC} \cdot A = J_{SC} w_{cell} l_{cell} = q \int_0^{\lambda_G} T(\lambda) \phi_{AM1.5G}(\lambda) d\lambda \; w_{cell} l_{cell} \tag{S14}$$

where $T(\lambda)$ is the wavelength dependence transmission spectrum of the TE, λ_G is the band gap of the semiconductor (Si is used in this work: $\lambda_G = 1100$ nm), and $\phi_{AM1.5G}(\lambda)$ is the AM1.5G solar spectrum flux. The dark current is determined by the black body radiation photon flux $\phi_{BB}(\lambda)$ of the device at its temperature of 300 K, and is given by:

$$I_0 = J_0 \cdot A = J_0 w_{cell} l_{cell} = q \int_0^{\lambda_G} T(\lambda) \phi_{BB}(\lambda) d\lambda \; w_{cell} l_{cell} \tag{S15}$$

According to Planck's law of black body radiation, the black body photon flux as is given by:

$$\phi_{BB}(\lambda, T = 300 \text{ K}) = \frac{2\pi c}{\lambda^4} \frac{1}{\exp\left(\frac{hc}{\lambda k_B T} - 1 \right)} \tag{S16}$$

where c is the speed of light, and h is the Planck constant.

The reader is referred to the work of Anand et al. for more details of the calculation of the FoM.[10]

References

(1) Van De Groep, J.; Spinelli, P.; Polman, A. Transparent conducting silver nanowire networks . *Nano Letters* **2012**, *12*, 3138–3144.

(2) Kung, S. C.; Xing, W.; Donavan, K. C.; Yang, F.; Penner, R. M. Photolithographically patterned silver nanowire electrodeposition . *Electrochimica Acta* **2010**, *55*, 8074–8080.

(3) Sciacca, B.; van de Groep, J.; Polman, A.; Garnett, E. C. Solution-Grown Silver Nanowire Ordered Arrays as Transparent Electrodes . *Advanced Materials* **2016**, *28*, 905–909.

(4) Patterson, A. L. The Scherrer Formula for X-Ray Particle Size Determination . *Physical Review* **1939**, *56*, 978–982.

(5) Ansys Lumerical, ANSYS, Inc.: Canonsburg, PA, USA; https://www.lumerical.com.

(6) Palik, E. D. *Handbook of Optical Constants of Solids* ; Academic Press, 1985.

(7) König, T. A. F.; Ledin, P. A.; Kerszulis, J.; Mahmoud, M. A.; El-Sayed, M. A.; Reynolds, J. R.; Tsukruk, V. V. Electrically Tunable Plasmonic Behavior of Nanocube–Polymer Nanomaterials Induced by a Redox-Active Electrochromic Polymer . *ACS Nano* **2014**, *8*, 6182–6192.

(8) Haacke, G. New figure of merit for transparent conductors . *Journal of Applied Physics* **1976**, *47*, 4086–4089.

(9) Dressel, M.; Grüner, G. *Electrodynamics of Solids: Optical Properties of Electrons in Matter* ; Cambridge University Press, 2002.

(10) Anand, A.; Islam, M. M.; Meitzner, R.; Schubert, U. S.; Hoppe, H. Introduction of a Novel Figure of Merit for the Assessment of Transparent Conductive Electrodes in Photovoltaics: Exact and Approximate Form . *Advanced Energy Materials* **2021**, *11*.
